# Evaluation of human enterovirus 71 and coxsackievirus A16 specific immunoglobulin M antibodies for diagnosis of hand-foot-and-mouth disease

**DOI:** 10.1186/1743-422X-9-12

**Published:** 2012-01-11

**Authors:** Nan Yu, Min Guo, Si-Jie He, Yu-Xian Pan, Xin-Xin Chen, Xi-Xia Ding, Wei Hao, Ya-Di Wang, Sheng-Xiang Ge, Ning-Shao Xia, Xiao-Yan Che

**Affiliations:** 1 Center for Clinical Laboratory, Zhujiang Hospital, Southern Medical University, No. 253 Gong ye da dao zhong, Guangzhou, Guangdong 510282, PR China; 2 Department of Pediatrics, Zhujiang Hospital, Southern Medical University, No. 253 Gong ye da dao zhong, Guangzhou, Guangdong 510282, PR China; 3 National Institute of Diagnostics and Vaccine Development in infectious Disease, No. 422 South Siming Road, Xiamen University, Xiamen, Fujian 361005, PR China

**Keywords:** Enterovirus, HEV71, CVA16, Hand-Foot-and-Mouth Disease, IgM-capture ELISA, Cross-reactivity

## Abstract

**Background:**

Hand-foot-and-mouth disease (HFMD) is caused mainly by the human enterovirus type 71 (HEV71) and the Coxsackievirus A group type 16 (CVA16). Large outbreaks of disease have occurred frequently in the Asia-Pacific region. Reliable methods are needed for diagnosis of HFMD in childen. IgM-capture ELISA, with its notable advantages of convenience and low cost, provides a potentially frontline assay. We aimed to evaluate the newly developed IgM-capture ELISAs for HEV71 and CVA16 in the diagnosis of HFMD, and to measure the kinetics of IgM over the course of HEV71 or CVA16 infections.

**Results:**

We mapped, for the first time, the kinetics of IgM in HEV71 and CVA16 infection. HEV71- and CVA16-IgM were both detectable in some patients on day 1 of illness, and in 100% of patients by day 5 (HEV71) and day 8 (CVA16) respectively; both IgMs persisted for several weeks. The IgM detection rates were 90.2% (138 of 153 sera) and 68.0% (66 of 97 sera) for HEV71 and CVA16 infections, respectively, during the first 7 days of diseases. During the first 90 days after onset these values were 93.6% (233 of 249 sera) and 72.8% (91 of 125 sera) for HEV71 and CVA16 infections, respectively. Some cross-reactivity was observed between HEV71- and CVA16-IgM ELISAs. HEV71-IgM was positive in 38 of 122 (31.1%) CVA16 infections, 14 of 49 (28.6%) other enteroviral infections and 2 of 105 (1.9%) for other respiratory virus infected sera. Similarly, CVA16-IgM was apparently positive in 58 of 211 (27.5%) HEV71 infections, 16 of 48 (33.3%) other enterovirus infections and 3 of 105 (2.9%) other respiratory virus infected sera. Nevertheless, the ELISA yielded the higher OD_450 _value of main antibody than that of cross-reaction antibody, successfully identifying the enteroviral infection in 96.6% (HEV71) and 91.7% (CVA16) cases. When blood and rectal swabs were collected on the same day, the data showed that the agreement between IgM-capture ELISA and real-time RT-PCR in HEV71 was high (Kappa value = 0.729) while CVA16 somewhat lower (Kappa value = 0.300).

**Conclusions:**

HEV71- and CVA16-IgM ELISAs can be deployed successfully as a convenient and cost-effective diagnostic tool for HFMD in clinical laboratories.

## Background

Hand-foot-and-mouth disease (HFMD), characterized by fever and acute vesicular eruptions of palms, soles of the feet and mouth (herpangina), is a common exanthema in young children. It is caused by members of the non-polio Enterovirus genus (family *Picornoviridae)*, such as Coxsackievirus A (CVA) and B, Echovirus 4, 6 and 7, particularly CVA16 and human enterovirus (HEV) 71. Outbreaks have occurred recently in the Asia-Pacific region: Malaysia (2000-2003) [1], Taiwan (1998-2005) [2,3], Singapore (2000) [4], Brunei (2006) [5], Thailand (2008-2009) [6], Korea (2008-2009) [7], and Hong Kong (2008) [8]. In mainland China, large epidemics of HFMD have been reported: Shenzhen (1999-2004) [9], Beijing (2008) [10], and Fuyang city (2008) [11]. Surveillance studies have indicated that HEV71 and CVA16 circulate widely in central and southern China. The severe complications and even fatal cases in young children associated with HEV71 make HFMD an important health concern. With large outbreaks occurring frequently and the increased concern of fatal HFMD caused by HEV71, a rapid, specific, and cost-effective assay to identify the HFMD-causing enterovirus is of great importance. Recognition of the causative agent for HFMD mainly relies on laboratory identification of the virus so that treatment and effective public health measures can be taken early.

Diagnostic techniques include time consuming and labor intensive methods such as virus isolation, a neutralization test, and RT-PCR for viral RNA detection. In contrast, newly developed IgM-capture ELISAs for HEV71 [12,13] and CVA16 [14] are rapid and convenient for large numbers of specimens. Previously, capture ELISAs for HEV71- and CVA16-IgM were established, which show good efficiency for screening HFMD patients [12,14]. An understanding of the kinetic profiles of the IgM antibodies and the diagnostic characteristic of these assays is needed to substantiate their validity. In this study, we aimed to evaluate IgM-capture ELISAs for HEV71 and CVA16 for diagnosis of HFMD in pediatric patients, and to follow the kinetics of IgM antibodies over the course of these infections.

## Materials and methods

### Patients and clinical samples

HFMD patients with clinic features of herpangina, aseptic meningitis, and encephalitis, hospitalized in Zhujiang Hospital from March 2009 to December 2010, were studied. Laboratory diagnosis of all these patients showed them to be infected with HEV71, CVA16 or other enteroviruses as detected on rectal swabs using real-time RT-PCR plus virus isolation in some cases. Selected cases were confirmed by the neutralization test. The assay results showed 134 HFMD patients (86 male and 48 female, aged 4 months to 14.1 years, median 2.17 years) with HEV71 infection, 67 HFMD patients (49 male and 18 female, aged 6 months to 7.0 years, median 2.17 years) with CVA16 infection, and 29 HFMD patients (21 male and 8 female, aged 5 months to 5.6 years, median 1.83 years) with other enteroviral infections. A total of 434 acute- and convalescent-phase serum specimens were collected between days 1 and 158 after the onset of symptoms from these 230 HFMD patients (a single sample from 69 patients, two from 139 individuals, three from 18 patients, two from four individuals and six from one patient). Nineteen consecutive sera from one patient, confirmed as infected with HEV71 by real-time RT-PCR in combination with virus isolation, were assayed for HEV71-IgM during the course of the disease.

As controls, 105 sera from 75 patients with acute respiratory infections were collected. All these patients had been laboratory-confirmed previously as being infected with respiratory syncytial virus (RSV, 40 patients), adenovirus (9), influenza A virus (5), influenza B virus (2), parainfluenza virus (5), human rhinovirus (3), human metapneumovirus (3) and other respiratory viruses (8) by real-time RT-PCR and/or virus isolation.

### Real-time RT-PCR and VP1 semi-nested RT PCR

Viral RNA extraction was performed on swab specimens using the QIAamp Viral RNA Mini Kit (Qiagen). Real-time RT-PCR was performed in a Lightcycler 1.2 (Roche) using Pan-Enterovirus-, HEV71- and CVA16-specific detecting kits (Da An Gene Co., Ltd.). After 25 min of reverse transcription at 40°C and denaturation at 94°C for 3 min, 40 cycles of amplification (denaturation: 93°C, 15 sec; annealing/elongation: 55°C, 45 sec) were used. The semi-nested RT-PCR was as described previously [15] using RNA extracted from rectal swabs. Sequencing of the amplified VP1 gene product identified the serotype.

### Virus Isolation

Viral isolation was attempted on selected rectal swabs that were real-time RT-PCR positive. After shaking vigorously and centrifugation (4°C, 10,000 × g, 20 min), samples were sterilized by filtration (0.22 μm Millipore express^® ^membrane) and used to inoculate human rhabdomyosarcoma (RD) and/or laryngeal carcinoma cells. Once a complete cytopathic effect (CPE) was noted, cultures were harvested and viral identification was performed by real-time RT-PCR as described above.

### Neutralization test

HEV71 and CVA16 specific neutralizing antibodies were detected according to a standard protocol [16]. Briefly, 50 μl two-fold serially diluted serum was mixed with an equal volume of HEV71 or CVA16 (100 TCID_50_/50 μl) and incubated at 34°C for 2 h. The mixtures were added to replicate microplate cultures of RD cells and incubated at 34°C for 7 days. CPE was observed under a microscope after 2 to 7 days. The highest dilution that prevented the occurrence of the CPE was designated as the neutralizing antibody titer.

### IgM-capture ELISA

The IgM-capture ELISA for HEV71 and CVA16 has been described previously [12,14]. The cutoff value was set as 0.1 plus mean OD_450 _value of negative controls. An S/CO (sample/cutoff) value greater than 1.0 indicated a positive result.

### Statistical analysis

Sensitivity and specificity were calculated from the ELISA and real-time RT-PCR results. Differences between the proportions of positive results were compared by McNemar's chi-square test using SPSS software (version.13.0) and considered to be significant when *P *< 0.05.

## Results

### Kinetics of IgM antibodies in HEV71 and CVA16 infections

**Figure 1:**
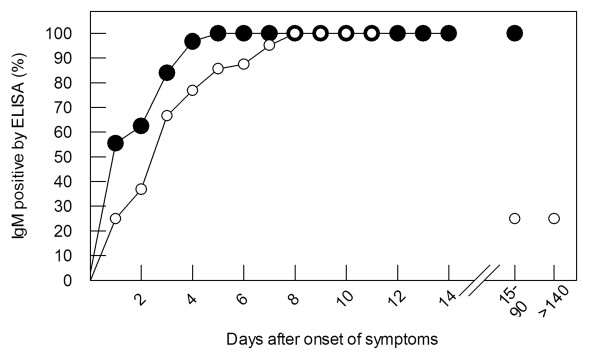
**The positive rate of detecting the HEV71- (●) and CVA16-specific (○) IgM as a function of time after the onset of symptoms**.

To measure the kinetics of IgM antibodies, serum samples were obtained on day 1 to 158 after onset from 134 HEV71 infected patients (256 serum samples) and 67 CVA16 infected patients (129 serum samples) and were tested using HEV71- and CVA16-IgM ELISAs. HEV71- and CVA16-IgM were both detectable on day 1 of illness and persisted for several weeks (Figure 1). The 100% positive rate was reached at day 5 (HEV71) and day 8 (CVA16) respectively. By twenty weeks after onset, the IgM detection rate had decreased substantially. None of seven HEV71 sera taken after 90 days of disease were HEV71-IgM positive and only one of four CVA16 sera taken after 90 days of disease was weakly positive for CVA16-IgM (S/CO value = 1.09).

The overall sensitivity was 90.2% (138 of 153 sera) for HEV71 and 68.0% (66 of 97 sera) for CVA16 infections during the first 7 days and 93.6% (233 of 249 sera) for HEV71 and 72.8% (91 of 125 sera) for CVA16 infections during the first 90 days of disease (Table 1). The positive rate of HEV71-IgM was significantly higher than that of CVA16-IgM during the first week (*P *= 0.000), and also over the first 90 days of disease (*P *= 0.000). One HEV71 infected patient, who provided 19 consecutive sera, tested positive for HEV71-IgM from day 5 to 74. Antibody remained high (S/CO>7.0) until day 43, then declined (S/CO: 1.5-4.3).

**Table 1:** The positive detection rate of HEV71- and CVA16-IgM antibodies by the IgM-capture ELISA.

	No. of positive/No. of sera				Positive predictive value (95% confidence interval)	Negative predictive value (95% confidence interval)
			
IgM-capture ELISAs	HEV71 infected sera	CVA16 infected sera	Other enterovirus infected sera	Respiratory virus infectious sera		
HEV71-IgM	233/249	38/122	14/49	2/105	81.3% (76.9-85.1%)	91.0% (87.5-93.8%)

CVA16 -IgM	58/211	91/125	16/48	3/105	54.4% (48.3-60.9%)	88.6% (85.1-91.4%)

Note: The data were collected during the first 90 days of disease.

### Cross-reactivity of HEV71- and CVA16-IgM ELISAs

To measure the specificity of the two IgM-capture ELISAs, HEV71-IgM was measured in 122 sera from 67 CVA16 patients and 49 sera from 29 other enterovirus infected patients. Similarly, CVA16-IgM was measured in 211 sera from 116 HEV71 patients and 48 sera from 29 other enterovirus infected patients. We also measured both IgMs in 105 sera from 75 respiratory virus infected patients to further test the specificity. Serotyping by semi-nested RT-PCR [15] of the 29 other enterovirus infected patients showed CVA6 in four individuals, CVA10 in two, one each of CVA21, CVB2, Echo 16 or Echo 25, and 19 untyped; the latter were further confirmed as neither HEV71 nor CVA16 infected by the neutralization test. HEV71-IgM was positive in 38 of 122 CVA16, 14 of 49 other enterovirus and two (both from one patient co-infected with RSV subgroup A and B) of 105 respiratory virus infected sera. The specificity was 69.6% (52/171) compared to other enteroviruses and 98.1% (2/105) compared to other respiratory viruses (Table 1). CVA16-IgM was apparently positive in 58 of 211 HEV71, 16 of 48 other enterovirus and 3 (from two patients co-infected with RSV subgroup A and B and one with RSV subgroup B) of 105 respiratory virus infected sera, giving a specificity of 71.4% (74/259) compared to other enteroviruses and 97.1% (3/105) compared to other respiratory viruses. The positive and negative predictive values were 81.3% (95% confidence interval: 76.9-85.1%) and 91.0% (95% confidence interval: 87.5-93.8%) for HEV71, 54.4% (95% confidence interval: 48.3-60.9%) and 88.6% (95% confidence interval: 85.1-91.4%) for CVA16, respectively (Table 1).

**Figure 2:**
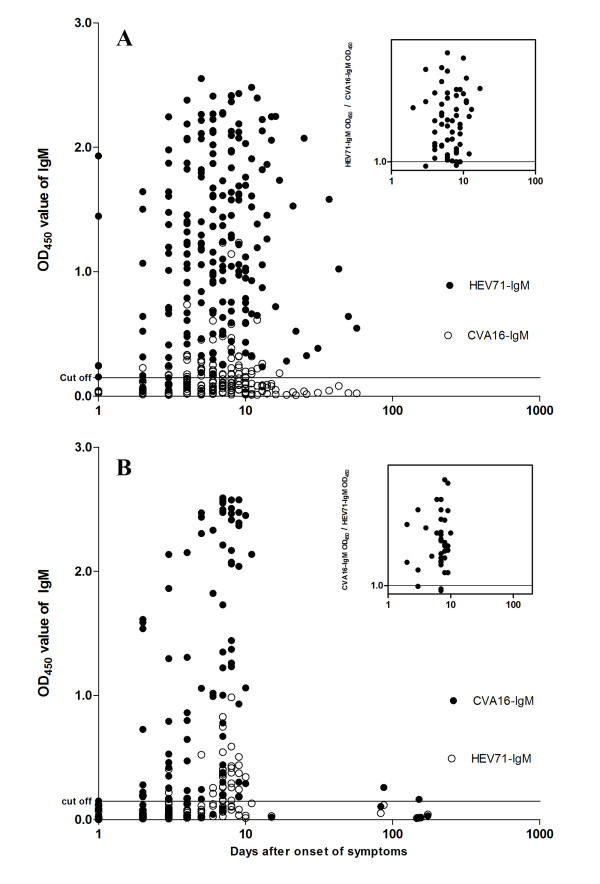
**OD_450 _values and ratio for HEV71-IgM and CVA16-IgM in HEV71 or CVA16 infected sera collected at various days after onset of symptoms**. Panel **A **shows the OD_450 _values for HEV71 infected patients; values greater than cutoff (horizontal line) represent a positive result. The inset shows the ratio of OD_450 _values for HEV71-IgM divided by that for CVA16-IgM. Panel **B **shows the OD_450 _values for CVA16 infected patients; values greater than 1.0 (horizontal line) represent a positive result. The inset shows the ratio of OD_450 _values for CVA16-IgM divided by that for HEV71-IgM.

To further analyze the cross-reactivity of the assays, a total of 206 sera from 134 HEV71 patients and 119 sera from 66 CVA16 infected patients were tested for both HEV71- and CVA16-IgM simultaneously (Figure 2). For the HEV71-infected patients (Figure 2A), 199 of 206 samples were positive for HEV71-IgM (95.7%) while the cross-reactivity towards CVA16-IgM was 28.2% (58/206). However, of the 58 CVA16-IgM positive sera, the ratio of OD_450 _value for HEV71-IgM divided by that for CVA16-IgM greater than 1.0 can successfully identify 56 (96.6%) HEV71 infections (Figure 2A, inset). For the CVA16 patients (Figure 2B), CVA16-IgM was detected in only 83 of 119 samples (69.7%) while cross-reactivity towards HEV71-IgM was seen in 30.3% (36/119). While of the 36 HEV71-IgM positive sera, the ratio of OD_450 _value for CVA16-IgM divided by that for HEV71-IgM greater than 1.0 can successfully identify 33 (91.7%) CVA16 infections (Figure 2B, inset).

### Correlation between real-time RT-PCR and IgM-capture ELISA results

In all patients, HEV71 or CVA16 was identified by real-time RT-PCR of a rectal swab, but this was not always taken on the same day that serum was collected. Subsets of the data, where both samples (111 HEV71 and 53 CVA16) were collected within 24 hours of one another were analyzed. For HEV71, the difference between ELISA and real-time RT-PCR was not statistically significant (McNemar's chi-square test exact *P *= 0.648) and the measure agreement was high (Kappa value = 0.729). For CVA16, the difference between ELISA and real-time RT-PCR was not statistically significant (McNemar's chi-square test exact *P *= 0.885) but the measure agreement was relatively low (Kappa value = 0.300). In other respects, the data are similar to those in Figure 1 in that IgM increased during the very early acute phase and reached the 100% positive rate by day 4 (HEV71) or 8 (CVA16). The sensitivity for HEV71-IgM was 90.1% (95% confidence interval: 84.1-94.4%) while that for CVA16-IgM was 56.6% (95% confidence interval: 44.5-67.9%).

## Discussion

Conventional methods for diagnosis of HEV71 and CVA16 infection (virus isolation, neutralization or RT-PCR) are slow, complex and/or costly, do not lend themselves to large number of specimens and are, therefore, unsuited to the clinics of developing countries. IgM-capture ELISA, with its notable advantages of convenience and low cost, provides a potentially frontline assay for diagnosis of HFMD.

We mapped, for the first time, the kinetics of IgM in HEV71 and CVA16 infection. In 138/153 sera of HEV71 and 66/97 sera of CVA16, IgM was detected during the acute phase (within 7 days after symptom onset), consistent with Wang' s study [13] for HEV71. The positive rate reached 100% at day five and eight, somewhat later than that of nucleic acid detection of HEV71 in throat and fecal samples from HFMD patients. However, the IgM is maintained for several months while the detection rate of nucleic acid fell markedly during 9-12 days after onset of disease [17]. For example, IgM was detected by Wang on day 94, while we found two cases, one each of HEV71 and CVA16 infection, where the corresponding IgM was detectable on day 74 and 87 respectively. However, 3-4 months after onset, both IgMs had largely declined to undetectable levels. Nevertheless, it should be noted that these results were obtained from multiple individuals and need to be confirmed using consecutive specimens from individual patients.

Recent results may be compared with those reported previously. The sensitivity for HEV71 (93.6%) is consistent with that reported (94.1%) [12], while that for CVA16 IgM (72.8%) was somewhat lower than that found earlier (84.6%) [14]. The discrepancy may due to the time when the sera were collected; our results show that CVA16 IgM is detectable in only 68% of patients in days 1-7 of illness, but rises to 100% on days 8-11. When blood and rectal swabs were collected on the same day, the agreement between capture-ELISA and real-time RT-PCR in both HEV71 and CVA16 infections suggested both capture-ELISAs perform as well as RT-PCR in diagnosing HFMD and could be deployed successfully in clinical and public health laboratories. Because the sample size was relatively small, particularly for CVA16, it is difficult to compare the sensitivity results with our larger data set.

We observed significant cross-reactivity between HEV71- and CVA16-IgM ELISAs and several reasons can be advanced for this apparent lack of specificity. First, co-infection by the two viruses could occur, leading to simultaneous production of both HEV71- and CVA16-IgMs. This is ruled out by the real-time RT-PCR results, which never detected both of these two viruses. Second, there may have been prior infection with the other virus. If this prior infection had been several months before clinical presentation, the dominant immunoglobulin isotype would be IgG, with the level of IgM low or undetectable. More recent prior infection could be the explanation; although the virus itself would have been cleared and not detected, the corresponding IgM can persist for several weeks. In this case, it would be expected that the cross-reactive IgM would have been detected in the earliest samples that were collected. Figure 2 shows that cross-reactivity is delayed, taking a few days to become evident.

The third hypothesis, which we favor, is that the IgMs may recognize a common epitope between these two related viruses. Homology between HEV71 and CVA16 is 77% at the genome level and 89% for amino acid sequences [18]. The resulting antigenic similarity means that infection with one virus could elicit antibodies against a second enterovirus serotype. This hypothesis is supported by the observed cross-reactivity with other enteroviruses (Table 1).

For example, 38 of 122 (31.1%) CVA16 infected samples were positive for HEV71-IgM, a value comparable to the 14 of 49 (28.6%) samples from other enteroviral infections. In contrast, only 2 of 105 (1.9%) respiratory virus infected sera were HEV71-IgM positive. This is strong evidence against the hypothesis that this cross-reactivity is due to a recent prior infection to HEV71. It seems unlikely that about 30% of the patients infected with CVA16 or with other enteroviruses were previously infected by HEV71, while only 2% of the respiratory virus infected patients had this prior infection. Similarly, CVA16-IgM was apparently positive in 58 of 211 (27.5%) HEV71 infected samples, 16 of 48 (33.3%) of other enterovirus infections, but only 3 of 105 (2.9%) for other respiratory virus infected sera. It was demonstrated, by virus neutralization tests, that none of the patients infected with other enteroviruses or other respiratory viruses, was virus-positive for HEV71 or CVA16.

We suggest that infection with either HEV71 or CVA16 results in several IgMs, some that are specific for the infecting virus and others that cross-react with related enteroviruses. From a practical standpoint, ELISA yielding the higher OD_450 _value was successful in identifying whether the enteroviral infection was by HEV71 or CVA16 in most cases. This is an important result because it can be used as a predictor to distinguish these two causes HFMD. A small proportion of HEV71-infected children develop severe and sometimes fatal neurological and systemic complications over days or even hours [19] so early diagnosis of the infecting virus is crucial.

## Conclusions

This study represents the first report of the kinetics of IgM in HEV71 and CVA16 infections. The IgM-capture ELISAs for HEV71 and CVA16 were found to be highly effective in correctly identifying the infecting virus. ELISAs have the advantage over RT-PCR to provide a convenient and relatively rapid diagnostic tool for HFMD infections. Assaying for both HEV71-IgM and CVA1-IgM can be deployed successfully as a cost-effective diagnosis of HFMD in clinical and public health laboratories.

## Ethical approval

Approval was obtained from the Ethics Committee of Zhujiang Hospital. The parents of each subject gave informed written consent before collection of rectal swabs and serum samples.

## List of abbreviations used

HFMD: hand-foot-mouth disease; HEV: human enterovirus; HEV71: human enterovirus 71 type; CVA: coxsackievirus A group; CVA16: coxsackievirus A group 16 type; CVB: coxsackievirus B group; RSV: respiratory syncytial virus; CPE: cytopathic effect; ELISA: enzyme-linked immunosorbent assay; RT-PCR: reverse transcription polymerase chain reaction;VP1: structural viral proteins 1; OD: optical density.

## Competing interests

The authors declare that they have no competing interests.

## Authors' contributions

NY performed the real-time RT-PCR and VP1 RT-semi-nest-PCR assays, analyzed the data and drafted the manuscript. MG collected most serum samples and performed the capture ELISA assays for both HEV71 and CVA16 specific IgM. SH and YP jointly performed virus isolation and identification. XC made clinical diagnoses and helped in collecting the clinical samples. XD and WH jointly performed PCR for respiratory virus infected patients and collected the serum samples. YW partly performed the real-time RT-PCR assay. SG and NX optimized the capture ELISA for HEV71 and CVA16 specific IgM, respectively, and jointly analyzed the data. XC conceived and designed the study, and drafted the manuscript. All authors read and approved the final manuscript.
